# Impact of treatment for adolescent and young adults with essential thrombocythemia and polycythemia vera

**DOI:** 10.1038/s41375-025-02545-2

**Published:** 2025-03-12

**Authors:** Yan Beauverd, Jean-Christophe Ianotto, Kyaw Htin Thaw, Marta Sobas, Parvis Sadjadian, Natalia Curto-Garcia, Lee-Yung Shih, Timothy Devos, Dorota Krochmalczyk, Serena Galli, Maria Bieniaszewska, Ilona Seferynska, Mary Frances McMullin, Anna Armatys, Adrianna Spalek, Joanna Waclaw, Mihnea Tudor Zdrenghea, Laurence Legros, Francois Girodon, Krzysztof Lewandowski, Beatriz Bellosillo, Jan Samuelsson, Aitor Abuin Blanco, Pascale Cony-Makhoul, Angela Collins, Chloe James, Rajko Kusec, Marie Lauermannova, Maria Soledad Noya, Malgorzata Skowronek, Lukasz Szukalski, Anna Szmigielska-Kaplon, Marielle Wondergem, Iryna Dudchenko, Joanna Gora-Tybor, Kamel Laribi, Anna Kulikowska de Nałęcz, Jean-Loup Demory, Katell Le Dû, Sonja Zweegman, Carlos Besses Raebel, Radek C. Skoda, Stephane Giraudier, Martin Griesshammer, Jean-Jacques Kiladjian, Claire N Harrison

**Affiliations:** 1 Hematology Division and Faculty of Medicine, Geneva University Hospitals, University of Geneva, Geneva, Switzerland; 2 Centre Hospitalier Universitaire de Brest, Brest, France; 3 Haematology Department, Guy’s and St Thomas’ NHS Foundation Trust, London, United Kingdom; 4 Wroclaw Medical University, Wroclaw, Poland; 5 University Clinic for Hematology, Oncology, Hemostaseology and Palliative Care, Johannes Wesling Medical Center, University of Bochum, Minden, Germany; 6 Division of Hematology-Oncology, Chang Gung Memorial Hospital and Chang Gung University, Taoyuan, Taiwan; 7 Department of Hematology, University Hospitals Leuven and Department of Microbiology and Immunology, Laboratory of Molecular Immunology (Rega Institute), KU Leuven, Leuven, Belgium; 8 Department of Hematology, Collegium Medicum, Jagiellonian University, Krakow, Poland; 9 Department of Medical Oncology and Hematology, University Hospital Zurich, Zurich, Switzerland; 10 Hematology and Transplantation Department, Medical University and Clinical Center, Gdansk, Poland; 11 Institute of Hematology and Transfusion Medicine, Warsaw, Poland; 12 Haematology, Belfast City Hospital, Queen’s University Belfast, Belfast, United Kingdom; 13 Department of Hematology and Bone Marrow Transplantation, Medical University of Silesia, Katowice, Poland; 14 Iuliu Hatieganu University of Medicine and Pharmacy, Department of Hematology, Cluj-Napoca, Romania; 15 Hematology Department, AP-HP, University of Paris Saclay, Bicêtre Hospital, Paris, France; 16 Laboratory of Biological Hematology, University Hospital, Dijon, France; 17 Hematology and Bone Marrow Transplantation Department, University of Medical Sciences, Poznan, Poland; 18 Hematology Department, Hospital del Mar, Hospital del Mar Research Institute, Barcelona, Spain; 19 Hematology Department, University Hospital, Linkoping, Sweden; 20 Servicio de Hematología. Hospital Universitario Lucus Augusti, Lugo, Spain; 21 Department of Hematology, Annecy-Genevois Hospital, Pringy, France; 22 Department of Haematology, Norfolk and Norwich University Hospitals National Health Service Trust, Norwich, United Kingdom; 23 Biology of Cardiovascular Diseases, University of Bordeaux, INSERM, UMR1034 Pessac, France; 24 Department of Hematology, University Hospital Dubrava, School of Medicine, University of Zagreb, Zagreb, Croatia; 25 Institute of Hematology and Blood Transfusion, Prague, Prague, Czech Republic; 26 Hematology Department, University Hospital CHUAC, A Coruña, Spain; 27 Department of Hematology, Holy Cross Oncology Center, Kielce, Poland; 28 Department of Hematology, Collegium Medicum in Bydgoszcz, Nicolaus Copernicus University, Torun, Poland; 29 Department of Hematology, Medical University, Lodz, Poland; 30 Department of Hematology, Amsterdam University Medical Centers, Amsterdam, Netherlands; 31 Department of Internal Medicine with Respiratory Medicine Center, Academic and Research Medical institute, Sumy State University, Sumy, Ukraine; 32 Hematology Department, Le Mans Hospital, Le Mans, France; 33 Department of Hematology, State Hospital, Opole, Poland; 34 Department of Hematology, St. Vincent De Paul Hospital, Lille, France; 35 The Confluent, Private Hospital, Nantes, France; 36 Experimental Hematology, Department of Biomedicine, University Hospital and University, Basel, Switzerland; 37 Cellular Biology Department, INSERM UMRS 1131, St Louis Hospital, APHP, Paris, France; 38 Hopital Saint-Louis, Paris, France; 39 Guy’s and St. Thomas’ NHS Foundation Trust, London, ENG United Kingdom

**Keywords:** Myeloproliferative disease, Risk factors, Drug therapy

## Abstract

Essential thrombocythemia (ET) and polycythemia vera (PV) are rare in adolescent and young adult (AYA). These conditions, similar to those in older patients, are linked with thrombotic complications and the potential progression to secondary myelofibrosis (sMF). This retrospective study of ET and PV patients diagnosed before age 25 evaluated complication rates and impact of cytoreductive drugs on outcomes. Among 348 patients (278 ET, 70 PV) with a median age of 20 years, the of thrombotic events was 1.9 per 100 patient-years. Risk factors for thrombosis included elevated white blood cell count (>11 × 10^9^/L) (HR: 2.7, *p* = 0.012) and absence of splenomegaly at diagnosis (HR: 5.7, *p* = 0.026), while cytoreductive drugs did not reduce this risk. The incidence of sMF was 0.7 per 100 patient-years. *CALR* mutation (HR: 6.0, *p* < 0.001) and a history of thrombosis (HR: 3.8, *p* = 0.015) were associated with sMF risk. Interferon as a first-line treatment significantly improved myelofibrosis-free survival compared to other treatments or the absence of cytoreduction (*p* = 0.046). Although cytoreduction did not affect thrombotic event, early interferon use reduced sMF risk. These findings support interferon use to mitigate sMF risk in AYA ET and PV patients.

## Introduction

Essential thrombocythemia (ET) and polycythemia vera (PV) belong to the myeloproliferative neoplasms (MPN) category of the WHO classification[1, 2]. These disorders involve myeloid proliferation, presenting as thrombocytosis in ET, increased red cell mass in PV and varyingly mild leukocytosis, splenomegaly or general symptoms, and an elevated risk of arterial and venous thrombotic events. Additionally, there is a potential, albeit less common, progression to secondary myelofibrosis (sMF), acute leukemia (AL), and a reduction in life expectancy [3].

While ET and PV typically manifest in the sixth decade, approximately 20% of cases are diagnosed in individuals under 40, with limited available data on adolescent and young adult (AYA) patients [4–7]. Current recommendations and risk classifications for ET and PV, such as ELN [8] and IPSET-T [9], are primarily based on data from older patients. The primary objective in managing these conditions is thrombotic risk reduction, achieved through pharmacological cytoreduction. Hydroxycarbamide (HU), interferon (IFN), and anagrelide (ANA) are commonly employed as first-line treatments, with IFN uniquely demonstrating long-term disease modification potential, including allele burden reduction and the possibility of treatment discontinuation [10–12].

Our study aimed to explore long-term complications, evaluating the impact of treatment on thrombotic risk and sMF progression. This becomes particularly pertinent given the extended life expectancy of these patients spanning several decades.

## Material and method

### Patient recruitment and data collection

Members of the European Hematology Association (EHA) Specialized Working Group for Myeloproliferative Neoplasms (EHA SWG MPN) retrospectively included patients with a diagnosis of ET or PV before the age of 25 years. Patients lacking molecular driver (*JAK2*, *CALR*, *MPL*) testing at any point were excluded.

We retrospectively collected sex, date of birth, thrombotic and bleeding history, laboratory values at diagnosis, driver mutation (*JAK2*, *CALR*, *MPL*), cardiovascular risk factors (CVRF), symptom burden, treatment history (antiplatelet, anticoagulant and cytoreductive drugs) and evolution (thrombotic events, progression to sMF and AL, death). The European LeukemiaNet (ELN) risk category for ET and PV, along with the IPSET-thrombosis risk category for ET, were assessed at the time of diagnosis for each patient.

Informed consent was obtained in accordance with local ethical and legal requirements. The study was approved by the Ethics Committee of Brest University Hospital (B2023CE.07) and conducted in compliance with the Declaration of Helsinki.

### Outcomes and statistics

Primary outcomes were thrombosis-free survival (TFS) and myelofibrosis-free survival (MFS) and overall survival (OS) in the entire cohort, and for ET and PV patients separately, and impact of treatment on these outcomes. Secondary outcomes were identification of risk factors associated with thrombotic events and sMF.

Patient characteristics were reported descriptively. Categorical variables were expressed as proportions and continuous variables as median and interquartile range (IQR) and compared using the Chi-square test or Fisher exact test for categorial variable and Wilcoxon rank-sum test for median values. TFS, MFS and OS were estimated using the Kaplan–Meier method [13] and the log-rank test was used to compare outcome probabilities. A Cox regression model was performed for TFS and MFS to identify associations with sex, disease, driver mutation, white blood cell (WBC) count, platelet (PLT) count, lactate dehydrogenase (LDH) level, splenomegaly, thrombosis history and CVRF. Regarding the impact of cytoreductive drugs, patients were classified by the first cytoreductive treatment they received (for at least 18 months for MFS). A two-sided *p*-value < 0.05 was considered significant. Data analysis was performed using SPSS version 23.0 (IBM Corp., Armonk, NY).

## Results

### Patients

We included 348 patients (278 ET and 70 PV). 249 (72%) were females and 99 (28%) males with a median follow-up of 8.5 years (IQR: 4.0–14.8). Median age at diagnosis was 20 years (IQR: 18–23) in the entire cohort, 21 years (IQR: 18–23) for ET and 20 years (IQR: 16–23) for PV. Regarding driver mutations for ET patients, 147 (53%) had *JAK2* mutation, 43 (16%) were *CALR* and 3 (1%) *MPL*-mutated while 85 (30%) were triple negative (TN). Of the 85 TN ET patients, 64 (75%) underwent a bone marrow biopsy at diagnosis, confirming the diagnosis in 60, while insufficient material was available for an accurate diagnosis in the remaining 4 patients. All 70 PV patients were *JAK2* mutated. 43 patients (12%) had an history of thrombotic event before or at diagnosis and 46 (14%) had cardiovascular risk factors (CVRF). At diagnosis, ELN risk category was low for 304 patients (88%) and high for 43 patients (12%). IPSET-thrombosis risk category for ET was low for 122 patients (45%), intermediate for 110 patients (41%) and high for 37 patients (14%). Patient characteristics are summarized in Table 1.

**Table 1:** Patient characteristics, for the entire cohort, for ET and PV patients.

	ALL (*n* = 348)		ET (*n* = 278)		PV (*n* = 70)		*p*-value
	*n*	%	*n*	%	*n*	%	
Age at diagnosis (median, IQR/range)	20yo	18–23/2–25	21yo	18–23/2–25	20yo	16–23/2–25	0.320
Sex							<0.001
Male	99	28.4	62	22.3	37	52.9	
Female	249	71.6	216	77.7	33	47.1	
Mutational status							<0.001
*JAK2*	217	62.4	147	52.9	70	100.0	
Exon 14	212		147		65		
Allele burden (median, IQR) (data available for 58 pts)	22.4	14–22	15.8	10.7–25.5	32.5	22.5–44	<0.001
Exon 12	5		0		5		
*CALR*	43	12.3	43	15.5	0	0.0	
Type 1	14		14		0		
Type 2	7		7		0		
Unknown	22		22		0		
*MPL*	3	0.9	3	1.1	0	0.0	
TN	85	24.4	85	30.5	0	0.0	
Biochemical analysis							
Hemoglobin (g/L) (median, IQR) (data available for 288 pts)	141.5	131–153	139	130–143	170	134–240	<0.001
Hematocrit (%) (median, IQR) (data available for 269 pts)	42.9	39.3–45.7	41.5	39–44	52	48.3–57.4	<0.001
Hematocrit >45% (data available for 269 pts)	84	31.2	41	18.4	43	93.5	<0.001
White blood cell (×10^9^/ul) (median, IQR) (data available for 288 pts)	9	7.5–11.2	8.8	7.4–10.7	11.4	7.1–13.3	0.001
White blood cell >11 ×10^9^/ul (data available for 288 pts)	77	26.7	52	21.5	25	54.3	<0.001
Platelets (×10^9^/ul) (median, IQR) (data available for 293 pts)	874	621–1253	913	664–1304	628	355–919	<0.001
Platelet count >1000 ×10^9^/ul (data available for 293 pts)	106	36.2	99	40.4	7	14.6	<0.001
LDH above normal range (data available for 217 pts)	98	45.2	73	42	25	58.1	0.056
Splenomegaly by palpation (data available for 305 pts)	54	17.7	33	13.1	21	38.9	<0.001
Reason for consulting							<0.001
Thrombotic event	27	7.8	13	4.7	14	20.0	
Hemorrhage	0	0.0	0	0.0	0	0.0	
MPN-related symptoms	72	20.7	57	20.5	15	21.4	
Abnormal blood count	71	20.4	66	23.7	5	7.2	
Abnormal blood count during pregnancy	15	4.3	12	4.3	3	4.3	
Unknown	163	46.8	130	46.8	33	47.1	
MPN-related symptoms							
Plethoric face (data available for 296 pts)	10	3.4	0	0.0	10	19.6	<0.001
Pruritus (data available for 304 pts)	16	5.3	7	2.8	9	16.1	<0.001
Hyperviscosity (data available for 296 pts)	101	35.3	78	33.8	23	41.8	0.261
Constitutive symptoms (data available for 299 pts)	14	4.0	10	3.6	4	5.7	0.088
Fatigue (data available for 281 pts)	55	19.6	38	16.3	17	35.4	0.002
Sweets (data available for 281 pts)	5	1.8	1	0.4	4	8.3	<0.001
Vascular symptoms (data available for 318 pts)	34	10.7	30	11.6	4	6.7	0.263
CVRF (presence of any) (data available for 341 pts)	46	13.5	35	12.8	11	16.2	0.469
Smoking	32	9.4	26	9.5	6	8.8	0.859
High blood pressure	7	2.1	4	1.5	3	4.4	0.145
Dyslipidemia	1	0.3	1	0.4	0	0.0	1.000
Diabetes	2	0.6	0	0.0	2	2.9	0.039
Obesity	5	1.5	4	1.5	1	1.5	1.000
Thrombosis history (data available for 347 pts)	43	12.4	26	9.4	17	24.6	<0.001
Venous	31	8.9	21	7.6	10	14.5	0.070
BCS	11	3.2	6	2.2	5	7.2	
BCS/CVT	1	0.3	0	0.0	1	1.4	
CVT	10	2.9	7	2.5	3	4.3	
DVT	3	0.9	3	1.1	0	0.0	
PE	2	0.6	2	0.7	0	0.0	
PVT	2	0.6	1	0.4	1	1.4	
RVT	1	0.3	1	0.4	0	0.0	
SpVT	1	0.3	1	0.4	0	0.0	
Arterial	9	2.6	3	1.1	6	8.7	0.003
AMI	3	0.9	1	0.4	2	2.9	
Stroke	2	0.6	1	0.4	1	1.4	
TIA	4	1.2	1	0.4	3	4.3	
Unknown localisation	3	0.9	2	0.7	1	1.4	
Bleeding history (data available for 347 pts)	20	5.8	17	6.1	3	4.3	0.775
ELN risk							<0.001
Low	304	87.6	252	90.6	52	75.4	
High	43	12.4	26	9.4	17	24.6	
IPSET thrombosis (data available for 269 pts)							
Low	N/A		122	45.4	N/A		
Intermediate	N/A		110	40.9	N/A		
High	N/A		37	13.7	N/A		

*ALL* entire cohort, *AMI* acute myocardial infarction, *BCS* Budd-Chiari syndrome, *CVRF* cardiovascular risk factors, *CVT* cerebral vein thrombosis, *DVT* deep vein thrombosis, *ELN* European LeukemiaNet, *ET* essential thrombocythemia, *IPSET* International prognostic score of thrombosis for essential thrombocythemia, *IQR* interquartile range, *MPN* myeloproliferative neoplasms, *N/A* not applicable, *PE* pulmonary embolism, *pts* patients, *PV* polycythemia vera, *PVT* portal vein thrombosis, *RVT* renal vein thrombosis, *SpVT* splenic vein thrombosis, *TIA* transient ischemic attack, *TN* triple negative

### Treatments

Data regarding antithrombotic treatment (antiplatelet and/or anticoagulant) were available for 342/348 patients. Of them, 283 (83%) received antithrombotic treatment (in ET: 222 patients, 81%; in PV: 61 patients, 90%), 234 (68%) of them were on antiplatelet drugs only (in ET: 191 patients, 70%; in PV: 43 patients, 63%); 38 (11%) received anticoagulants (in ET: 25 patients, 9%; in PV: 13 patients, 19%); and 11 (3%) were on dual treatment (in ET: 6 patients, 2%; in PV: 5 patients, 7%). Meanwhile 59 (17%) were not treated with any antithrombotic drug (in ET: 52 patients, 19%; in PV: 7 patients, 10%).

Overall, 237 (68%) patients were treated with a cytoreductive drug (ET: 185 patients, 66.5%; PV: 52 patients, 74.3%). Median time from diagnosis to first cytoreductive drug initiation was 85 days (IQR: 1–955 days) in the entire cohort (ET: 76 days (IQR: 1–809 days); PV: 164 days (IQR: 0–1574 days)). Indications for cytoreductive treatment introduction were: elevated platelet count (>1000 × 10^9^/L): 96 patients (41%) (ET: 87 patients, 47%; PV: 9 patients: 17%); history of thrombosis: 32 patients (14%) (ET: 24 patients, 3%; PV: 8 patients: 15%); MPN-related symptoms: 12 patients (5%) (ET: 7 patients, 4%; PV: 5 patients, 10%): pregnancy in 3 patients (2%) (only ET), too frequent phlebotomy: 4 patients (2%) (all PV); or unknown/other reason in 90 patients (38%) (ET: 64 patients, 35%; PV: 26 patients, 50%). We investigated cytoreduction according to the ELN risk score. Of the high-risk patients, 39/43 patients (91%) received a cytoreductive drug and 4/43 patients (9%) did not. Of the low-risk patients, 197/304 (65%) were treated with a cytoreductive drug and 107/304 (35%) were not.

We investigated the different lines of treatment and reasons for drug modification during follow-up (Table 2). Of the 237 patients treated with a cytoreductive drug, 97 (41%) received only one line, 82 (35%) two lines and 58 (24%) patients three or more lines of treatment. As first line, HU (126 patients, 53%) was the most frequently prescribed drug, followed by IFN (55 patients, 23%), ANA (52 patients, 22%), or alternative drugs (4 patients, 2%). Overall, the reason for treatment modification, when data was available, was mainly due to intolerance (14%), resistance (8.6%), and pregnancy wish (6.2%). Even if intolerance was the first reason of treatment modification in all drug categories, it was more frequent in IFN treated patients (26.4%) in comparison with HU (11.7%), ANA (18%) or other (0%).

**Table 2:** Treatment by line and overall, and reason for treatment change to the next treatment line.

	No change			Intol		Resist		Wish Preg		Other/Unk		CHR	
	*n*	*n*	%	*n*	%	*n*	%	*n*	%	*n*	%	*n*	%
**1st line**													
HU	126	38	30.2	14	11.1	11	8.7	15	11.9	46	36.5	2	1.6
IFN	55	28	50.9	16	29.1	5	9.1	2	3.6	4	7.3	0	0.0
PEGα2a	33	19	57.6	8	24.2	3	9.1	1	3.0	2	6.1	0	0.0
IFNα2a	22	9	40.9	8	36.4	2	9.1	1	4.5	2	9.1	0	0.0
ROPEGα2b	0												
ANA	52	30	57.7	5	9.6	5	9.6	6	11.5	6	11.5	0	0.0
OTHER	4	0	0.0	0	0.0	0	0.0	0	0.0	3	75.0	1	25.0
**2nd line**													
HU	23	11	47.8	5	21.7	4	17.4	1	4.3	2	8.7	0	0.0
IFN	85	41	48.2	22	25.9	2	2.4	1	1.2	14	16.5	5	5.9
PEGα2a	50	22	44.0	12	24.0	2	4.0	1	2.0	9	18.0	4	8.0
IFNα2a	33	18	54.5	9	27.3	0	0.0	0	0.0	5	15.2	1	3.0
ROPEGα2b	2	1	50.0	1	50.0	0	0.0	0	0.0	0	0.0	0	0.0
ANA	28	15	53.6	6	21.4	3	10.7	0	0.0	4	14.3	0	0.0
OTHER	4	3	75.0	0	0.0	1	25.0	0	0.0	0	0.0	0	0.0
**3rd line**													
HU	14	10	71.4	0	0.0	0	0.0	1	7.1	3	21.4	0	0.0
IFN	23	16	69.6	5	21.7	0	0.0	0	0.0	2	8.7	0	0.0
PEGα2a	11	8	72.7	3	27.3	0	0.0	0	0.0	0	0.0	0	0.0
IFNα2a	12	8	66.7	2	16.7	0	0.0	0	0.0	2	16.7	0	0.0
ROPEGα2b	0	0											
ANA	9	3	33.3	5	55.6	0	0.0	0	0.0	1	11.1	0	0.0
OTHER	9	5	55.6	0	0.0	1	11.1	1	11.1	1	11.1	1	11.1
**Overall (regardless of treatment line)**													
HU	163	59	36.2	19	11.7	15	9.2	17	10.4	51	31.3	2	1.2
IFN	163	85	52.1	43	26.4	7	4.3	3	1.8	20	12.3	5	3.1
PEGα2a	94	49	52.1	23	24.5	5	5.3	2	2.1	11	11.7	4	4.3
IFNα2a	67	35	52.2	19	28.4	2	3.0	1	1.5	9	13.4	1	1.5
ROPEGα2b	2	1	50.0	1	50.0	0	0.0	0	0.0	0	0.0	0	0.0
ANA	89	48	53.9	16	18.0	8	9.0	6	6.7	11	12.4	0	0.0
Other	17	8	47.1	0	0.0	2	11.8	1	5.9	4	23.5	2	11.8

*ANA* anagrelide, *CHR* complete hematological response, *HU* hydroxycarbamide, *IFN* interferon, *IFNα2a* interferon-alfa-2a, *Intol* intolerance, *No* number, *PEGα2a* peginterferon-alfa-2a, *Resist* resistance, *ROPEGα2b* ropeginterferon-alfa-2b, *Unk* unknown, *WishPreg* wish for pregnancy

### Thrombosis

During the follow-up 44 patients presented 57 thrombotic events (venous: 40 (70%), arterial: 17 (30%)); 42 in ET (venous: 31 (74%), arterial: 11 (26%)); and 15 in PV (venous: 9 (60%), arterial: 6 (40%)). The risk of a thrombotic event was 1.9 per 100 patients-years for the whole cohort, 1.8 per 100 patient-years for ET and 2.0 per 100 patient-years for PV. Ten and 20-year probability of TFS was 86.8% (95% CI: 82.4–91.2%) and 78.8% (95% CI: 71.5–85.9%) for the entire cohort, 86.9% (95% CI: 81.9–91.9%) and 80.0% (95% CI: 71.8–88.2%) for ET and 84.4% (95% CI: 77.2–95.6%) and 76.3% (95% CI: 62.5–90.1%) for PV. There was no significant difference between ET and PV (*p* = 0.790). The median age at the first thrombotic event was 26 years (IQR: 23.2–29.5 years) in the entire cohort, 26 years (IQR: 13.9–28.8 years) in ET and 27 years (IQR: 22.8–30.4 years) in PV. Additionally, the median time from diagnosis to the first thrombotic event was 3.6 years (IQR: 2.2–8.7 years) in all patients, 4.0 years (2.2–8.2 years) in ET and 3.0 (IQR: 1.9–9.9 years) in PV. This time was shorter for patients with a previous history of thrombosis (median 3.0 years, IQR: 2.0–4.0 years) compared to those without a history of thrombosis (median 4.2 years, IQR: 2.1–9.4 years).

Then we investigated the impact of the ELN risk scores (for the entire cohort and ET and PV separately), and the IPSET-T score for ET. For the whole cohort, ET and PV ELN risk score was not prognostic of TFS. In the entire cohort, 10-year and 20-year TFS for low risk was 88.0% (95% CI: 83.6–92.4%) and 80.9% (95% CI: 73.9–87.9%) and for high-risk 77.8% (95% CI: 62.6–93%) and 77.8 (95% CI: 62.6–93.0%), *p* = 0.208 respectively. For ET, 10-year and 20-year TFS for low risk was 88.3% (95% CI: 83.3–93.3%) and 81.0% (95% CI: 72.4–89.6%) and for high-risk 73.2% (95% CI: 52.0–94.4%) and 73.2% (95% CI: 52.0–94.4%), *p* = 0.131. For PV, 10-years and 20-years TFS for low risk was 86.7% (95% CI: 76.3–97.1%) and 79.7% (95% CI: 66.3–93.1%) and for high-risk 84.6% (95% CI: 64.6–100%) and 84.6% (95% CI: 64.6–100%), *p* = 0.880. In contrast, IPSET-T score for ET was prognostic of TFS (*p* = 0.009); 10-year and 20-year TFS for low risk was 94.1% (95% CI: 89.3–98.9%) and 91.8 (95% CI: 85.4–98.2%), for intermediate 82.5% (95% CI: 73.3–91.7%) and 74.3% (95% CI: 56.5–92.1%) and for high-risk 77.1% (95% CI: 60.3–93.9%) and 65.3% (95% CI: 44.3–86.3%).

**Fig. 1:**
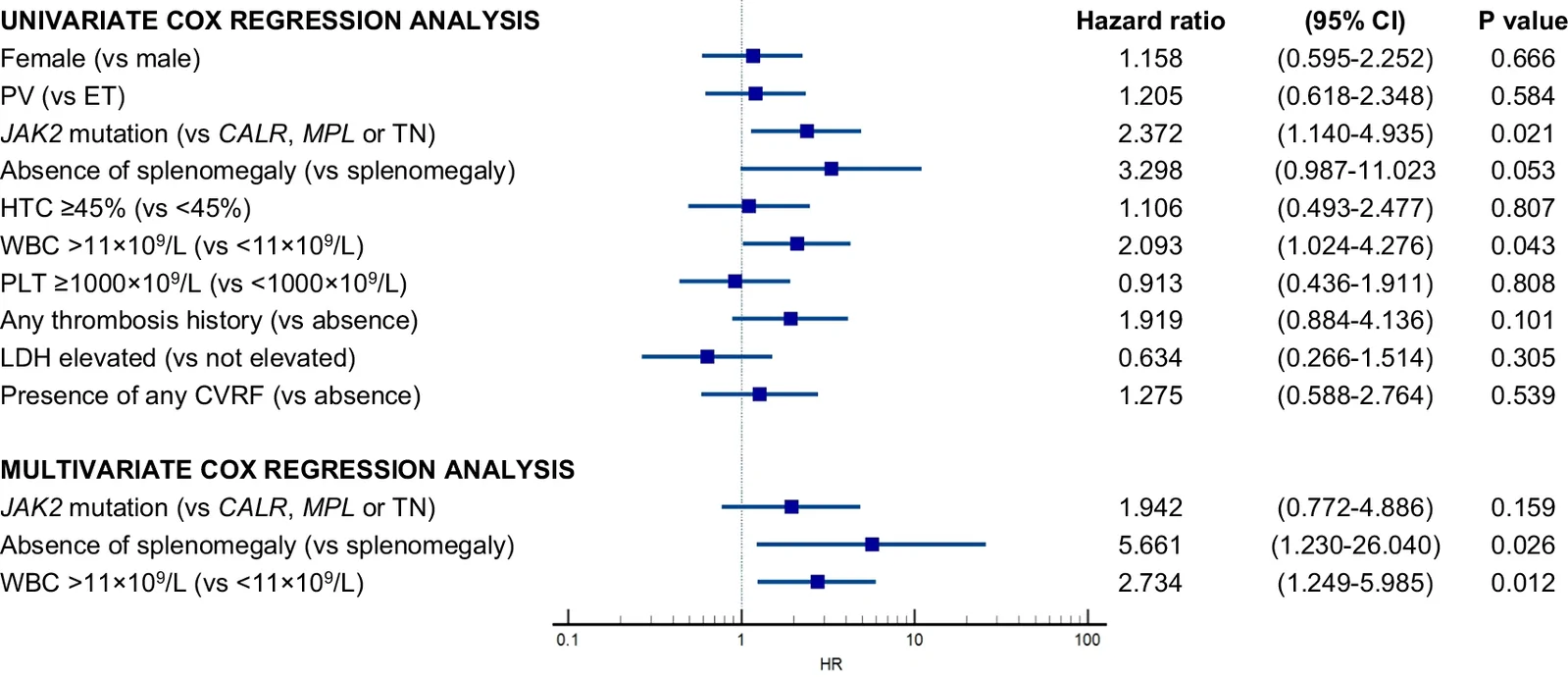
Univariate and multivariate analysis for risk factors associated with thrombotic risk in the entire cohort. CVRF cardiovascular risk factors, ET essential thrombocythemia, HCT hematocrit, LDH lactate dehydrogenase, PLT platelet, PV polycythemia vera, TN triple-negative, WBC white blood count.

Next, we investigated risk factors at diagnosis associated with the occurrence of thrombotic events (Fig. 1). In the entire cohort, in univariate analysis, *JAK2* mutation (HR:2.3, 95% CI: 1.2–4.8, *p* = 0.026), elevated WBC count (>11 × 10^9^/L) (HR: 2.1, 95% CI: 1.1–4.3, *p* = 0.043) and absence of splenomegaly at diagnosis (HR: 3.3, 95% CI: 1.0–11.0, *p* = 0.053) were associated with increased risk of thrombosis. In multivariate analysis, only elevated WBC count (HR: 2.7, 95% CI: 1.2–6.0, *p* = 0.012) and absence of splenomegaly at diagnosis (HR: 5.7, 95% CI: 1.2–26.0, *p* = 0.026) remained significant. For ET patients (Supplementary Fig. S1), only the presence of a *JAK2* mutation was associated with increased risk (HR: 2.6, 95% CI: 1.2–5.5, *p* = 0.012) in univariate analysis. For PV (Supplementary Fig. S1), none of the tested factors were able to predict the thrombotic risk. Finally, Supplementary Table S1 presents the frequency of thrombotic events, stratified by driver mutation.

Then, we investigated the impact of antiplatelet drugs on thrombotic events. In the entire cohort, in ET and in PV patients, antiplatelet drugs were not associated with a significant reduction in the risk of thrombosis (Supplementary Table S2). Next, we performed subgroup analysis investigating impact of antiplatelet therapy on high-risk patients as previously identified (*JAK2* mutated, elevated WBC count, absence of splenomegaly) but antiplatelet drugs were not associated with a better TFS either except for PV patients with elevated WBC (*p* = 0.046) and absence of splenomegaly (*p* = 0.017) (Supplementary Table S3)

**Fig. 2:**
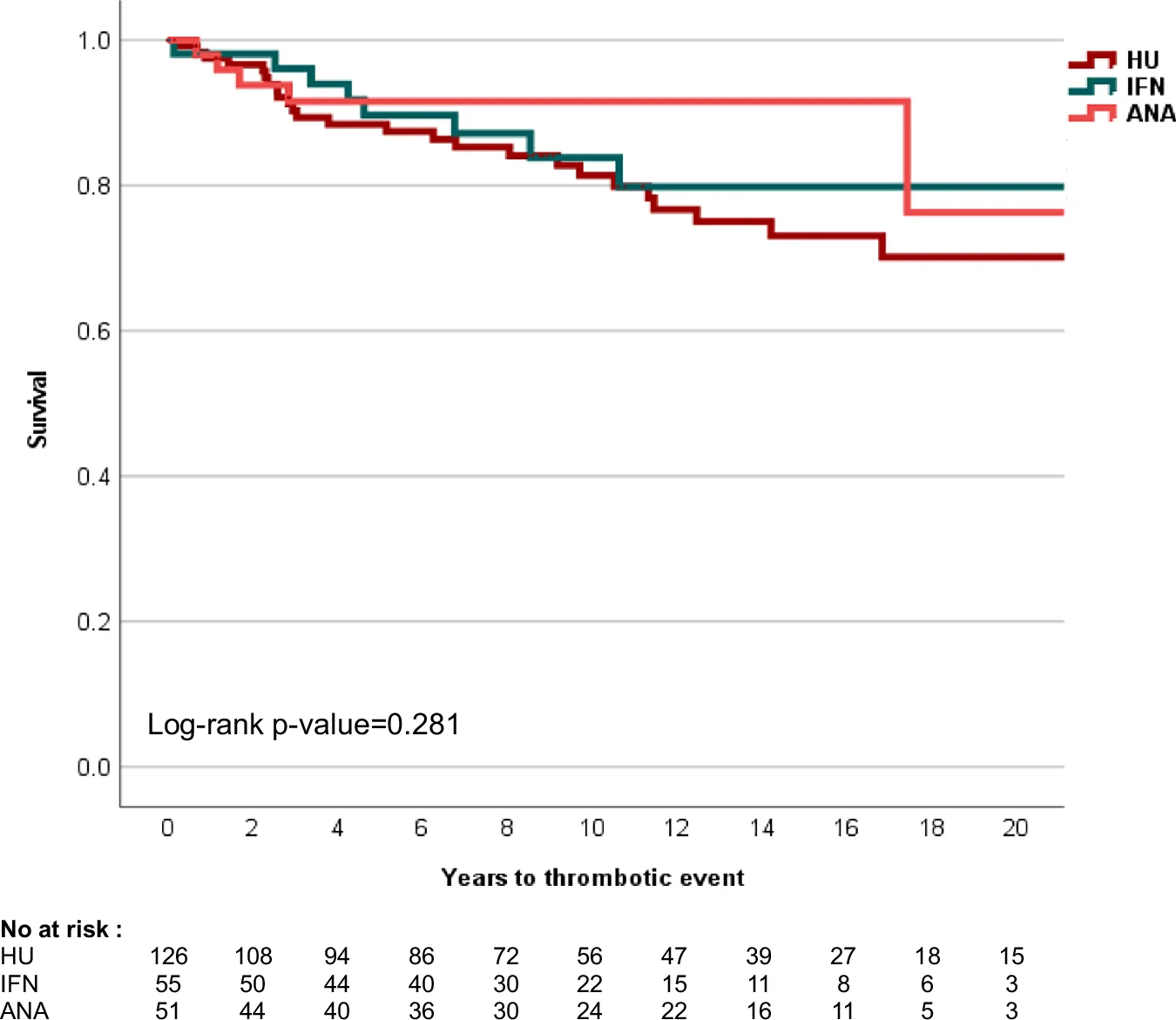
Thrombosis-free survival according to the first management (IFN vs. HU vs. ANA). ANA anagrelide, HU hydroxycarbamide, IFN interferon.

Finally, we investigated the impact of cytoreductive drugs choice in terms of TFS. For patients treated with any cytoreductive drugs, choice of first treatment was not associated with difference in terms of TFS. Indeed, for HU, 10-year and 20-year MFS were 81.4% (95% CI: 73.6–89.2%) and 70.2% (95% CI: 59–81.4%), for IFN they were 83.9% (95% CI: 72.3–95.5%) and 79.9% (95% CI: 66.5–93.3%) and for ANA they were 91.6% (95% CI: 83.6–99.6%) and 76.3% (95% CI: 47.7–100%) (*p* = 0.281) (Fig. 2). The results are similar when analyzing ET and PV patients separately (Supplementary Table S4, Supplementary Figure S3). Lastly, we conducted a subgroup analysis to examine the effect of cytoreduction in high-risk patients, (*JAK2* mutated, elevated WBC count, absence of splenomegaly). In this high-risk population either, there was no impact of cytoreduction on TFS (Supplementary Table S5).

### Myelofibrosis

During the follow-up, 22 progressions to sMF were observed, 18 in ET (6%) and 4 in PV (6%). Risk of MF progression was 0.7 per 100 patient-years in the entire cohort, 0.8 per 100 patient-years in ET and 0.5 per 100 patient-years in PV. Ten-year and 20-year MFS for the entire cohort was 95.2% (95% CI: 92.4–98%) and 81.3% (95% CI: 71.5–91.1%). For ET, 10-year MFS was 94.7% (95% CI: 91.2–98.3%) and 20-year MFS was 75.7% (95% CI: 61.9–89.5%). For PV, 10-year MFS was 96.8% (95% CI: 92.4–100%) and 20-year MFS was 93.3% (95% CI: 85.1–100). There was no significant difference between ET and PV (*p* = 0.236). The median time from diagnosis to sMF progression was 10 years (IQR: 5.2–17.1 years) in the entire cohort, 10 years (IQR: 5.7–17.1 years) in ET and 8.5 years (IQR: 2.4–19.5 years) in PV. The median age at sMF progression was 32 years (IQR: 27.4–40.6 years) for all patients, 33 years (IQR: 29–40.6 years) in ET and 27 years (IQR: 18.1–39.2) in PV.

**Fig. 3:**
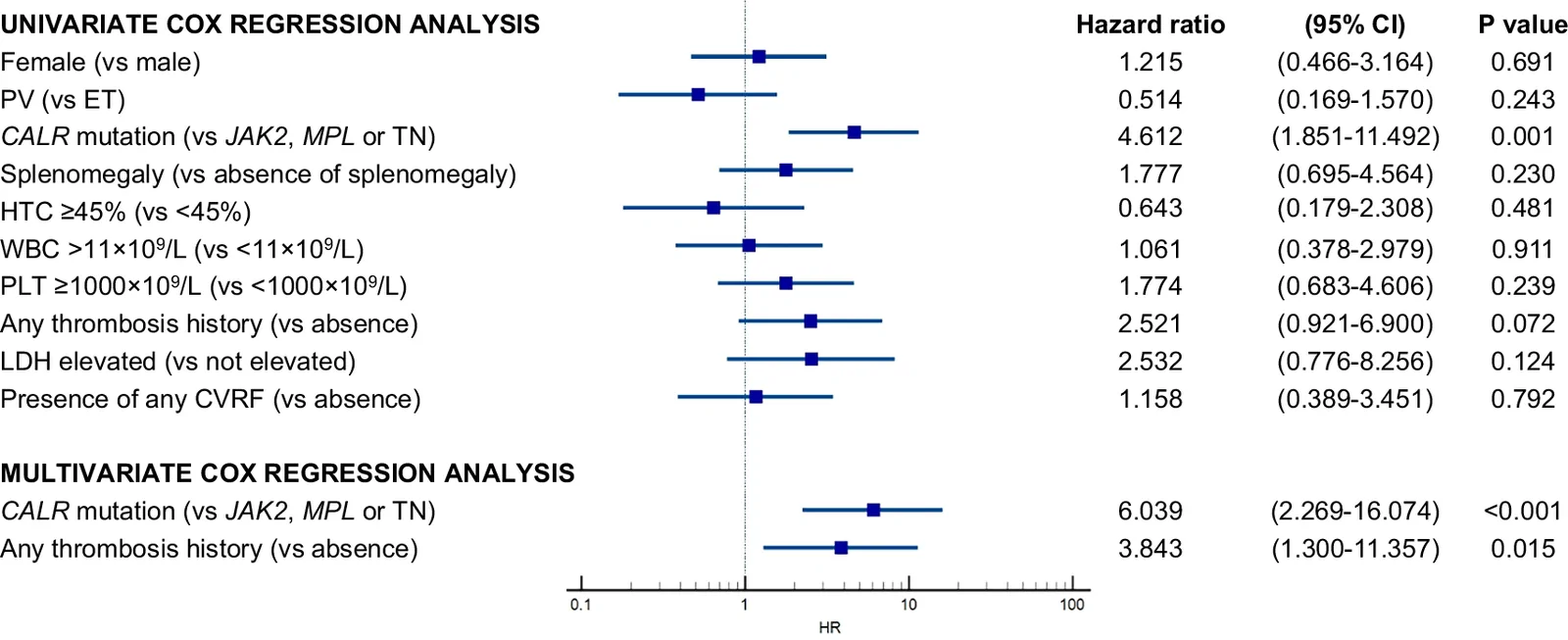
Univariate and multivariate analysis for risk factors associated with risk of myelofibrosis progression in the entire cohort. CVRF cardiovascular risk factors, ET essential thrombocythemia, HCT hematocrit, LDH lactate dehydrogenase, PLT platelet, PV polycythemia vera, TN triple-negative, WBC white blood count.

Concerning risk factors at diagnosis for sMF progression in univariate analysis, presence of *CALR* mutation (HR: 4.6 [95% CI: 1.9–11.5], *p* = 0.001) was associated with increased risk of sMF progression. Thrombosis history (HR: 2.5 [95% CI: 0.9–6.9], *p* = 0.072) showed a trend in the same direction. In multivariate analysis, *CALR* (HR: 6.0 [2.3–16.1], *p* < 0.001) and thrombosis history (HR: 3.8 [95% CI: 1.3–11.4], *p* = 0.015) were both significant. Results of univariate and multivariate analysis for the entire cohort are presented in Fig. 3. For ET, only *CALR* mutation (HR: 4.1 [95% CI: 1.6–10.5], *p* = 0.004) was significant. No risk factors were identified in PV. Univariate analysis for ET and PV are presented in the Supplementary Fig. S2 and frequency of myelofibrosis progression, stratified by driver mutation are presented in the Supplementary Table S1.

**Fig. 4:**
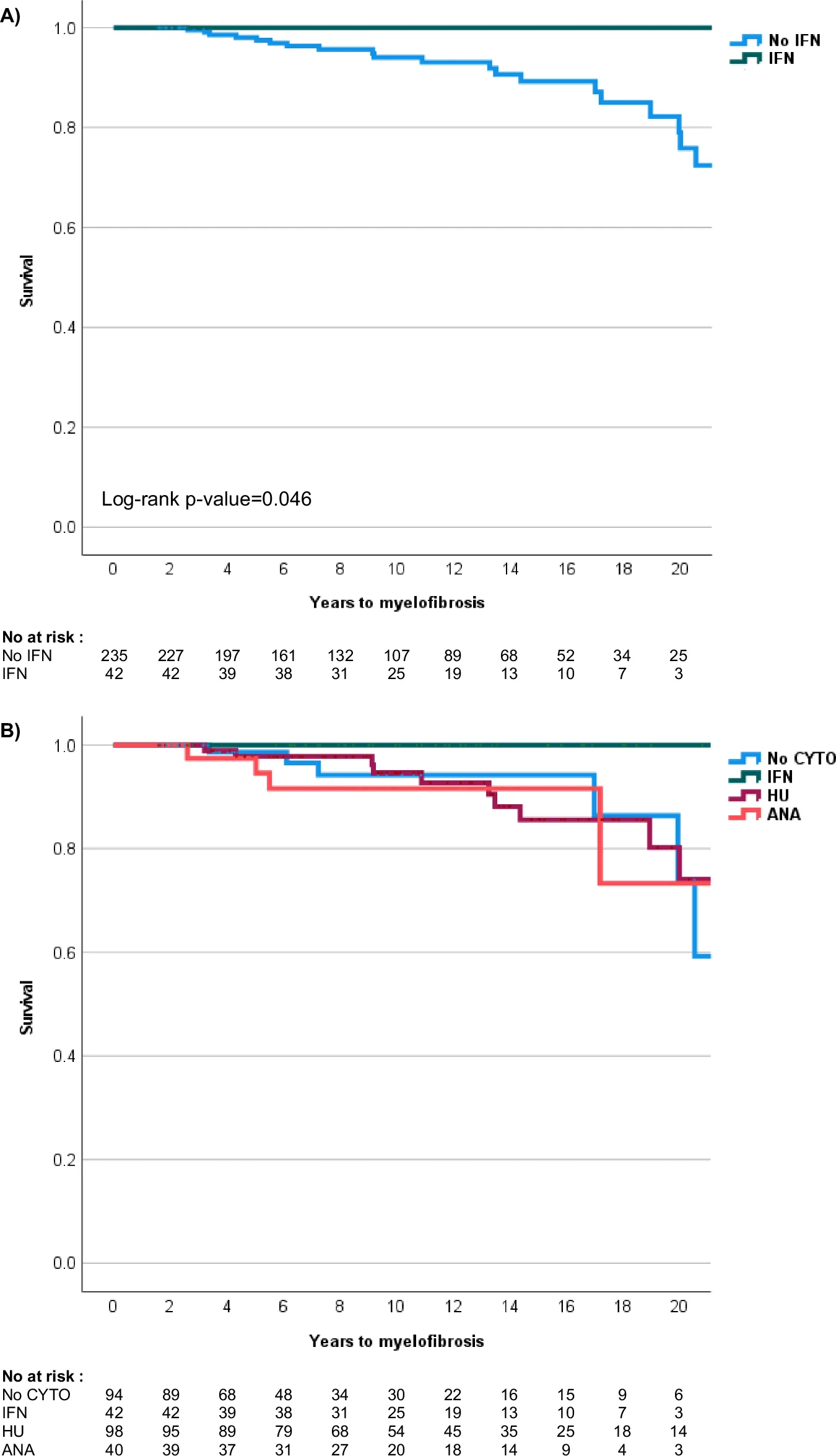
Myelofibrosis-free survival. **A** According to the first management (No IFN vs. IFN). **B** According to the first management (No CYTO vs. IFN vs. HU vs. ANA). ANA anagrelide, HU hydroxycarbamide, IFN interferon, No CYTO no cytoreduction, No IFN No interferon.

**Fig. 5:**
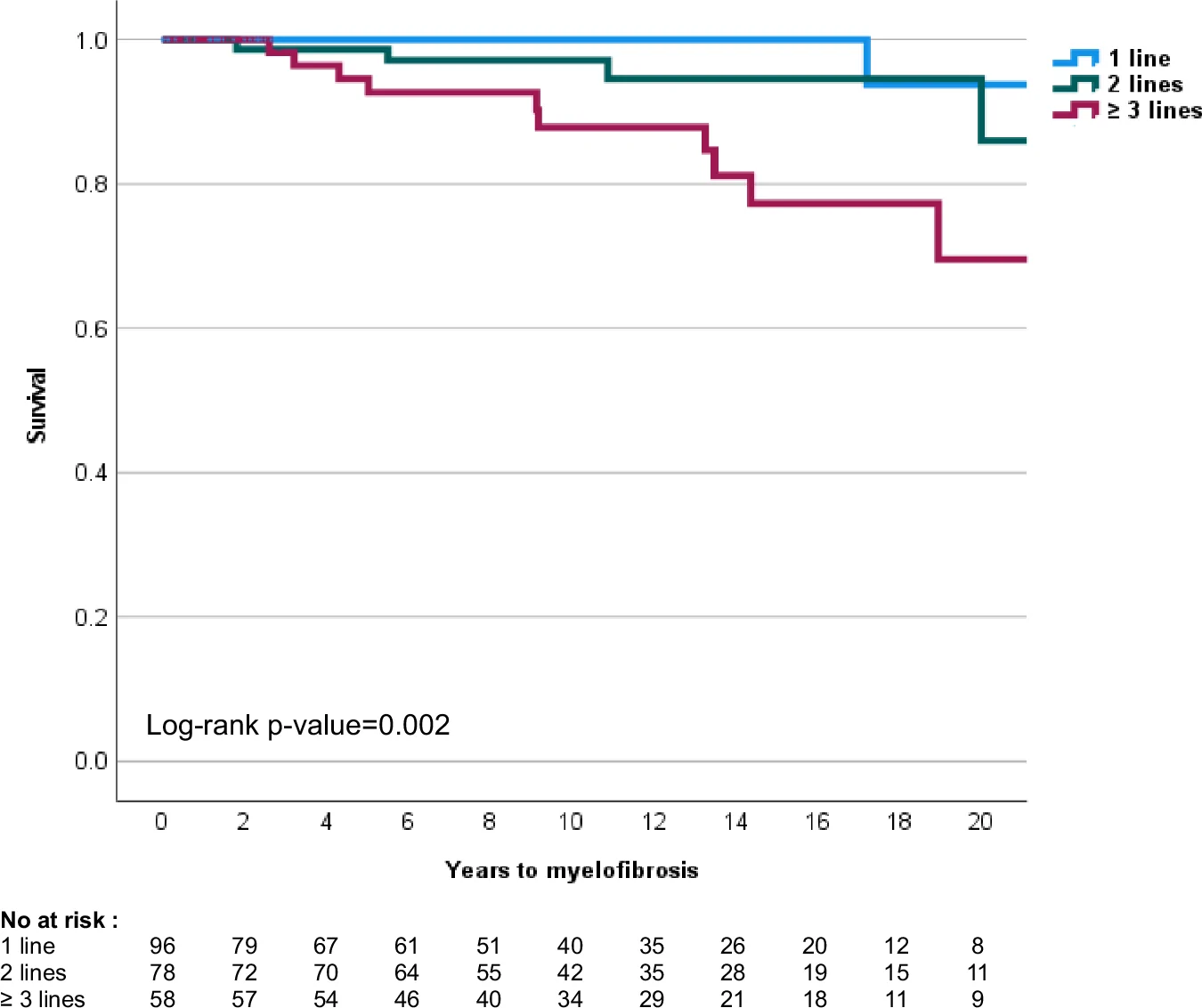
Myelofibrosis-free survival according to the number of lines of cytoreduction.

For the impact of first cytoreductive therapy on sMF progression, 10-year and 20-year MFS were both 100% for IFN. For HU, 10-year and 20-year MFS were 92.7% (95% CI: 86.3–99.1%) and 74.1% (95% CI: 56.7–91.5%), for ANA they were 91.6% (95% CI: 82.4–100%) and 73.3% (95% CI: 39.7–100%) and for No CYTO they were 94.2 (95% CI: 87.6–100%) and 74.0% (95% CI: 47.2–100%). When comparing IFN with other therapies (HU, ANA or No CYTO), probability of sMF progression was significantly lower in patients treated with IFN (*p* = 0.046) (Fig. 4A, B). When specifically analyzing ET and PV patients, the results were similar, with progression to myelofibrosis observed only in those not treated with IFN. The results are presented in Supplementary Table S6 and Supplementary Fig. S4. Finally, we investigated the impact of multiple lines of cytoreductive drugs on MFS. For patients on any cytoreduction, risk of sMF progression was higher for patients with two lines of treatments (HR: 4.0 [0.4–35.4], *p* = 0.219) and for patients on three or more line (HR: 12.9 [1.7–99.8], *p* = 0.014) when compared with patient treated with only one line (Fig. 5).

### Myelodysplasia, acute leukemia and death

Finally, during the follow-up there was only one progression to myelodysplastic syndrome, no patient developed AL and four patients died (1 of graft-versus-host disease following hematopoietic stem cell transplantation 34 years after diagnosis, 1 of CMV disease 15 years after diagnosis, and 2 of unknown cause 4 and 41 years after diagnosis).

Ten and 20-year probabilities of overall survival were 99.5% (95% CI: 98.5–100%) and 98.3% (95% CI: 95.7–100%) for the entire cohort, 100% and 98.3% (95% CI: 94.9–100%) for ET, and 98% (95% CI: 94–100%) and 98% (95% CI: 94–100%) for PV. There was no significant difference between patients with ET and PV (*p* = 0.789).

## Discussion

In this study, we focused on the largest well-defined cohort of individuals diagnosed with ET and PV before the age of 25. Our investigation focused on the risks of thrombosis, evolution to sMF, and the impact of treatment modalities.

Surprisingly, our findings revealed a significant thrombotic risk despite young age, with a rate of 1.9 per 100 patient-years, irrespective of the MPN subtype. This rate is comparable to those reported in contemporary cohorts of older patients with more comorbidities, although the time to the first thrombotic event appears to be longer in our cohort (median time of 4 years for ET and 3 years for PV) compared to adult cohorts (median time of 0.9 years for ET and 0.3 years for PV) [14–17]. Nevertheless, given that the incidence of thrombotic events appears to be as frequent in AYA patients as in older individuals, it remains a significant concern. These patients are expected to live for several decades, and the accumulation of additional thrombotic risk factors (age, cardiovascular conditions, additional mutations) may progressively heighten this risk. This highlights a substantial and emerging concern regarding thrombotic events in this younger demographic.

Unexpectedly, conventional risk scores, such as the ELN risk score [8, 18], failed to predict thrombosis in our AYA cohort. Elevated WBC count (>11 × 10^9^/L) emerged as an independent risk factor for thrombotic events (HR: 2.7, 95% CI: 1.2–6.0, *p* = 0.012) in multivariate analysis, consistent with findings in older patient cohorts [19, 20]. In contrast, splenomegaly demonstrated a protective effect in terms of thrombotic risk in multivariate analysis (HR: 0.2, 95% CI: 0.1–0.8, *p* = 0.026), contrary to previous publications [21]. Our study demonstrated a statistical association between splenomegaly and a reduced risk of thrombotic events. However, this study was not designed to establish causality, and we can speculate that this association reflects the influence of external factors rather than a direct causal relationship. For instance, splenomegaly was more frequently observed in patients with splanchnic thrombosis (42%) compared to those without splanchnic thrombosis (16%) (*p* = 0.004), which may indicate portal hypertension rather than myeloproliferative neoplasms is responsible for the splenomegaly. Additionally, patients with a history of splanchnic thrombosis are typically on long-term anticoagulation therapy, unlike others, which could partly explain the observed difference in thrombotic risk among patients with splenomegaly. Unfortunately, the data from our study cannot directly address this question. While a *JAK2* mutation showed an association with increased thrombotic risk in univariate analysis (HR:2.3, 95% CI: 1.2–4.8, *p* = 0.026), it was no longer significant in multivariate analysis. The impact of the presence of the *JAK2*V617F mutation has been well established in ET as a thrombotic risk factor. However, the allele burden is also a risk factor, and its implication could not be evaluated in this study due to incomplete data from our cohort. Moreover, the allele frequency of the *JAK2*V617 mutation in our cohort is lower than the data reported in the literature for older patients [22], particularly for those with PV, which could provide insight into the similar thrombotic risk between ET and PV patients. The absence of a correlation between previous thrombosis history and CVRF within our cohort of AYA patients diverges from the typical risk factors identified in their older counterparts. We hypothesize that these differences can have distinct reasons. Firstly, in our AYA cohort, thrombotic events in unusual sites —such as Budd-Chiari Syndrome or other splanchnic vein thrombosis, cerebral vein thrombosis—constitute the majority (53.5%) of thrombotic occurrences at the time of diagnosis. This starkly contrasts with their relative rarity in older patient populations, suggesting unique pathophysiological mechanisms in AYA patients. Specifically, interactions between activated blood cells and the splanchnic endothelial environment appear to be unique to splanchnic vein thrombosis in this age group [23, 24]. Secondly, CVRF, often associated with atherosclerotic plaques as a common cause of thrombotic events, have a distinct profile in AYA patients. In our cohort, the prevalence of CVRF was notably low (13.5%) and consisted mainly of active tobacco use. Given that atherosclerosis typically necessitates decades to manifest as a chronic disease [25], it is not surprising that CVRF are not predictive of thrombotic events within the relatively short timeframe of AYA patient observations. This underscores the imperative for a nuanced understanding of thrombotic risk factors tailored to different age groups.

While identifying risk factors at diagnosis remains crucial for pinpointing patients prone to thrombotic complications, within our cohort, we are unable to demonstrate a discernible advantage of cytoreduction to prevent thrombosis and absence of advantage of specific agent (HU, IFN or ANA) to mitigate this risk. In light of our results, the early use of pharmacological cytoreduction solely for the purpose of reducing thrombotic risk must be weighed against the risks and complications associated with the treatment, as we did not demonstrate any benefit from its use. Given the retrospective nature of our study, data regarding blood cell counts (specifically hematocrit, PLT count, and WBC count) over time and at the time of thrombotic complications were not systematically available, precluding us from determining whether the lack of efficacy of cytoreduction in reducing thrombotic risk was related to poorly controlled blood cell counts as previously demonstrated in older populations [14, 26–28], or to the ineffectiveness of cytoreduction in the AYA patients despite achieving target blood cell counts values. Finally, in light of our results, the use of antiplatelet therapy, has not shown a benefit in reducing thrombotic risk. However, the lack of evidence for a reduction in TFS with antiplatelet therapy may be related to the high use of these treatments in the cohort (75% in the overall cohort, 79% in ET, and 86% in PV). In the absence of more robust results, the use of antiplatelet therapy appears reasonable in AYA PV and ET patients, as recommended in the older population. This avenue warrants further investigation to refine our understanding and optimize therapeutic strategies in AYA patients.

In AYA patients, progression to sMF is a clear concern with a risk of 0.7 per 100 patient-years in our cohort, which is not different from the estimated rate in older cohorts [29]. Moreover, the median time between diagnosis and progression to sMF in our AYA patient cohort appears to be similar to the data published for older patient cohorts, suggesting that this risk is a particular concern for these younger patients [30–32]. Regarding risk factors for sMF progression, *CALR* mutation and thrombotic history were identified as independent risk factors in our population. Our data is in line with other studies in older patients [33–35]. However, due to the retrospective nature of our study, data on the type of *CALR* mutation (type 1 or type 2) and the allele frequency of mutations were available only for few patients. Given that literature suggests that type 1 *CALR* mutations and a high allele burden are factors associated with shorter MFS, our results should be interpreted with caution and should be investigated in AYA patients in future studies [36, 37]. Additionally, we investigated the impact of treatment in terms of MFS. Interestingly, our data suggest that early treatment specifically with IFN, as a first-line option, is associated with significantly better MFS compared to other management approaches (absence of cytoreductive drug or alternative cytoreduction). Although our study suffers from various biases, such as the lack of randomization, the retrospective nature of the study, or the use of more than one line of treatment in 59% of the patients, these results are consistent with recent retrospective [38] or prospective publications [39] and underscore the importance of IFN in the long-term control of MPN^31^. Furthermore, similarly to other publications [29], our study demonstrated that the more patients are exposed to a significant number of treatment lines, the greater the risk of progression to sMF. Our study does not allow us to establish a causal relationship between the number of treatment lines and the risk of sMF progression, but it is, at the very least, a risk factor for such a complication and should alert the physician to the possibility of such a complication.

Ideally, randomized trials should provide higher-quality data, however, studies investigating IFN, such as MPD-RC [11], PROUD-PV, CONTINUATION-PV [39] or DALIAH [40] studies lack a specific focus on AYA patients. Furthermore, the follow-up periods in these trials are limited, preventing a comprehensive assessment of long-term outcomes, such as MFS. The present study possesses distinctive strengths, notwithstanding its inherent limitations as a retrospective investigation, and the predominance of patients with ET vs PV. Firstly, the inclusion of only patients with a mutation work-up at diagnosis allowed us to restrict our cohort to an MPN population and exclude cases of poorly characterized polycythemia or thrombocytosis at diagnosis. Secondly, the inclusion of patients from 38 sites across 15 countries enhances the reproducibility of our results. Thirdly, a substantial proportion of patients in our cohort received initial treatment with IFN, enabling a robust analysis of MFS associated with IFN.

The study underscores important limitations in applying adult risk stratification algorithms and treatment guidelines to AYA patients with PV and ET [41]. Unlike adults, AYA patients naturally face a lower age-related risk and have a different range of cardiovascular risk factors. Although thrombosis rates are similar, the benefits of antiplatelet therapy are not as well established in AYA patients. Crucially, the high risk of disease progression in this population highlights the need for therapies that can address this risk. Notably, no progression was observed in AYA patients treated with interferon, strongly supporting its potential as a first-line treatment for this younger group, who are expected to live with their MPN for many years.

## Supplementary information

Supplemental material

## Data Availability

The datasets generated during and/or analyzed during the current study are available from the corresponding author on reasonable request.
