# Supplementary material for: Impact of treatment for adolescent and young adults with essential thrombocythemia and polycythemia vera
Source: Leukemia. 2025 Mar 12;39(5):1135–45. doi: 10.1038/s41375-025-02545-2 (PMC12055580; doi:10.1038/s41375-025-02545-2)
Supplement: Supplementary file 1 — Supplemental material [file 41375_2025_2545_MOESM1_ESM.pdf]

Table S1. Frequency of thrombotic events and myelofibrosis progression during follow-up by driver mutation

**ENTIRE COHORT**				
	**JAK2 [n, (%)]**	**CALR [n, (%)]**	**MPL [n, (%)]**	**TN [n, (%)]**
**Thrombotic complication**				
No	**182 (83.9)**	**40 (93)**	**2 (66.7)**	**80 (94.1)**
Yes	**35 (16.1)**	**3 (7)**	**1 (33.3)**	**5 (5.9)**
**MF progression**				
No	**207 (95.4)**	**36 (83.7)**	**3 (100%)**	**80 (94.1)**
Yes	**10 (4.6)**	**7 (16.3)**	**0 (0)**	**5 (5.9)**
**ET PATIENTS**				
**Thrombotic complication**				
No	**124 (84.3)**	**40 (93)**	**2 (66.7)**	**80 (94.1)**
Yes	**23 (15.6)**	**3 (7%)**	**1 (33.3%)**	**5 (5.9)**
**MF progression**				
No	**141 (95.9)**	**36 (83.7)**	**3 (100)**	**4 (94.1)**
Yes	**6 (4.1)**	**7 (16.3)**	**0 (0)**	**5 (6.5)**
**PV PATIENTS**				
**Thrombotic complication**				
No	**58 (82.9)**	N/A	N/A	N/A
Yes	**12 (17.1)**	N/A	N/A	N/A
**MF progression**				
No	**66 (94.3)**	N/A	N/A	N/A
Yes	**4 (5.7)**	N/A	N/A	N/A

ET: essential thrombocythemia, MF : myelofibrosis PV: polycythemia vera

Table S2: 10-year and 20-year probability of thrombosis-free survival according to the use of antiplatelet therapy in the entire cohort, for ET and PV patients.

TFS in the entire cohort	No pts	No Tx	10-years	95%CI min	95%CI max	20-years	95%CI min	95%CI max	*p*-value
No antiplatelet therapy	59	2	93.8	85.4	100.0	93.8	85.4	100.0	0.325
Any antiplatelet therapy	234	21	91.0	86.6	95.4	85.6	79.4	91.8	
**TFS in ET patients**			**10-years**	**95%CI min**	**95%CI max**	**20-years**	**95%CI min**	**95%CI max**	***p*-value**
No antiplatelet therapy	52	1	96.0	88.2	100.0	96	88.2	100.0	0.151
Any antiplatelet therapy	191	18	89.2	83.6	94.8	85.2	78.2	92.2	
**TFS in PV patients**			**10-years**	**95%CI min**	**95%CI max**	**20-years**	**95%CI min**	**95%CI max**	***p*-value**
No antiplatelet therapy	7	1	83.3	52.9	100.0	83.3	52.9	100.0	0.354
Any antiplatelet therapy	43	3	97.4	92.2	100.0	88.9	76.5	100.0	

No pts: number of patients, No Tx: number of thrombotic events, TFS: thrombosis-free survival

Table S3: 10-years and 20-years probability of thrombosis-free survival for high-risk patients (*JAK2*V617F, elevated WBC, absence of splenomegaly)

TFS for JAK2 (all patients)	No Pts	No Tx	10-years	95%CI min	95%CI max	20-years	95%CI min	95%CI max	*p*-value
No antiplatelet therapy	27	1	94.4	83.6	100	94.4	83.6	100	0.330
Any antiplatelet therapy	140	6	88.0	81.2	94.8	80.9	71.7	90.1	
**TFS for JAK2 (ET patients)**			**10-years**	**95%CI min**	**95%CI max**	**20-years**	**95%CI min**	**95%CI max**	***p*-value**
No antiplatelet therapy	20	0	100	100	100	100	100	100	0.119
Any antiplatelet therapy	97	13	82.3	72.1	92.5	76.8	64.6	89.0	
**TFS for JAK2 (PV patients)**			**10-years**	**95%CI min**	**95%CI max**	**20-years**	**95%CI min**	**95%CI max**	***p*-value**
No antiplatelet therapy	7	1	83.3	52.9	100	83.3	52.9	100	0.354
Any antiplatelet therapy	43	3	97.4	92.2	100	88.9	76.5	100	
**TFS for elevated WBC (all patients)**			**10-years**	**95%CI min**	**95%CI max**	**20-years**	**95%CI min**	**95%CI max**	***p*-value**
No antiplatelet therapy	13	1	87.5	64.1	100	87.5	64.1	100	0.968
Any antiplatelet therapy	48	5	93.2	85.6	100	79.7	60.9	98.5	
**TFS for elevated WBC (ET patients)**			**10-years**	**95%CI min**	**95%CI max**	**20-years**	**95%CI min**	**95%CI max**	***p*-value**
No antiplatelet therapy	10	0	100	100	100	100	100	100	0.315
Any antiplatelet therapy	33	5	89.6	78.2	100	70.6	45.0	96.2	
**TFS for elevated WBC (PV patients)**			**10-years**	**95%CI min**	**95%CI max**	**20-years s**	**95%CI min**	**95%CI max**	***p*-value**
No antiplatelet therapy	3	1	66.7	12.3	100	66.7	12.3	100	0.046
Any antiplatelet therapy	15	0	100	100	100	100	100	100	
**TFS for no splenomegaly (all patients)**			**10-years**	**95%CI min**	**95%CI max**	**20-years**	**95%CI min**	**95%CI max**	***p*-value**
No antiplatelet therapy	48	1	96.0	88.2	100	96.0	88.2	100	0.269
Any antiplatelet therapy	169	13	89.8	83.8	95.8	87.9	81.1	94.7	
**TFS for no splenomegaly (ET patients)**			**10-years**	**95%CI min**	**95%CI max**	**20-years**	**95%CI min**	**95%CI max**	***p*-value**
No antiplatelet therapy	44	0	100	100	100	100	100	100	0.071
Any antiplatelet therapy	147	13	87.9	80.9	94.9	85.8	77.8	93.8	
**TFS for no splenomegaly (PV patients)**			**10-years**	**95%CI min**	**95%CI max**	**20-years s**	**95%CI min**	**95%CI max**	***p*-value**
No antiplatelet therapy	4	1	66.7	12.3	100	66.7	12.3	100	0.017
Any antiplatelet therapy	22	0	100	100	100	100	100	100	

ET: essential thrombocythemia, PV: polycythemia vera, No pts: number of patients, No Tx: number of thrombotic events, TFS: thrombosis-free survival, WBC: white blood count

Table S4: 10-year and 20-year probability of thrombosis-free survival according to the use of cytoreductive therapy in the entire cohort, for ET and PV patients.

TFS in the entire cohort	No pts	No Tx	10-years	95%CI min	95%CI max	20-years	95%CI min	95%CI max	*p*-value
**HU**	126	26	81.4	73.6	89.2	70.2	59	81.4	0.281
**IFN**	55	8	83.9	72.3	95.5	79.9	66.5	93.3	
**ANA**	51	5	91.6	83.6	99.6	76.3	47.7	104.9	
**TFS in ET patients**			**10-years**	**95%CI min**	**95%CI max**	**20-years**	**95%CI min**	**95%CI max**	***p*-value**
**HU**	100	18	82.5	73.9	91.1	75.8	64.8	86.8	0.453
**IFN**	33	6	78.9	61.9	95.9	72.9	53.3	92.5	
**ANA**	50	5	91.4	83.2	99.6	76.2	47.6	100	
**TFS in PV patients**			**10-years**	**95%CI min**	**95%CI max**	**20-years**	**95%CI min**	**95%CI max**	***p*-value**
**HU**	26	8	77.6	59.8	95.4	60.3	37.9	82.7	0.372
**IFN**	22	2	90.4	77.4	100	90.4	77.4	100	
**ANA**	1	0	100	100	100	100	100	100	

ANA: anagrelide, IFN: interferon, HU: hydroxyurea, No pts: number of patients, No Tx: number of thrombotic events, TFS: thrombosis-free survival

Table S5: 10-years and 20-years probability of thrombosis-free survival for high-risk patients (*JAK2*V617F, elevated WBC, absence of splenomegaly)

TFS for JAK2 (all patients)	No Pts	No Tx	10-years	95%CI min	95%CI max	20-years	95%CI min	95%CI max	*p*-value
No cytoreduction	73	4	91.4	82.8	100	91.4	82.8	100	0.065
Any cytoreduction	144	31	79.7	72.1	87.3	68.8	57.8	79.8	
**TFS for JAK2 (ET patients)**			**10-years**	**95%CI min**	**95%CI max**	**20-years**	**95%CI min**	**95%CI max**	***p*-value**
No cytoreduction	55	2	92.9	82.5	100	92.9	82.5	100	0.051
Any cytoreduction	92	21	76.7	66.5	86.9	65.6	49.4	81.8	
**TFS for JAK2 (PV patients)**			**10-years**	**95%CI min**	**95%CI max**	**20-years**	**95%CI min**	**95%CI max**	***p*-value**
No cytoreduction	18	2	87.7	71.3	100	87.7	71.3	100	0.061
Any cytoreduction	52	10	84.6	73.6	95.6	72.7	56.7	88.7	
**TFS for elevated WBC (all patients)**			**10-years**	**95%CI min**	**95%CI max**	**20-years**	**95%CI min**	**95%CI max**	***p*-value**
No cytoreduction	21	2	86.3	67.9	100	86.3	67.9	100	0.576
Any cytoreduction	56	11	82.2	71.4	93	73	57.4	88.6	
**TFS for elevated WBC (ET patients)**			**10-years**	**95%CI min**	**95%CI max**	**20-years**	**95%CI min**	**95%CI max**	***p*-value**
No cytoreduction	14	1	90	71	100	90	71	100	0.667
Any cytoreduction	38	7	84.9	72.3	97.5	71.3	50.7	91.9	
**TFS for elevated WBC (PV patients)**			**10-years**	**95%CI min**	**95%CI max**	**20-years s**	**95%CI min**	**95%CI max**	***p*-value**
No cytoreduction	7	1	80	44.8	100	80	44.8	100	0.654
Any cytoreduction	18	4	75.9	54.7	97.1	75.9	54.7	97.1	
**TFS for no splenomegaly (all patients)**			**10-years**	**95%CI min**	**95%CI max**	**20-years**	**95%CI min**	**95%CI max**	***p*-value**
No cytoreduction	89	4	92.1	84.1	100	92.1	84.1	100	0.047
Any cytoreduction	162	27	81.8	74.6	89	73.3	60.7	85.9	
**TFS for no splenomegaly (ET patients)**			**10-years**	**95%CI min**	**95%CI max**	**20-years**	**95%CI min**	**95%CI max**	***p*-value**
No cytoreduction	79	3	93.3	85.3	100	93.3	85.3	100	0.085
Any cytoreduction	139	21	84.1	76.9	91.3	73.7	58.7	88.7	
**TFS for no splenomegaly (PV patients)**			**10-years**	**95%CI min**	**95%CI max**	**20-years s**	**95%CI min**	**95%CI max**	***p*-value**
No cytoreduction	10	1	85.7	62.3	100	85.7	62.3	100	0.311
Any cytoreduction	23	6	68.5	46.3	90.7	68.5	46.3	90.7	

ET: essential thrombocythemia, PV: polycythemia vera, No pts: number of patients, No Tx: number of thrombotic events, TFS: thrombosis-free survival, WBC: white blood count

Table S6: 10-year and 20-year probability of myelofibrosis-free survival according to the use of interferon or not in the entire cohort, for ET and PV patients.

MFS in the entire cohort	No pts	No Tx	10-years	95%CI min	95%CI max	20-years	95%CI min	95%CI max	*p*-value
**No IFN**	235	21	94.1	90.3	97.9	79	67.8	90.2	0.046
**IFN**	42	0	100			100			
**MFS in ET patients**			**10-years**	**95%CI min**	**95%CI max**	**20-years**	**95%CI min**	**95%CI max**	***p*-value**
**No IFN**	194	18	93.3	88.9	97.7	70	53.8	86.2	0.117
**IFN**	24	0	100	100	100	100	100	100	
**MFS in PV patients**			**10-years**	**95%CI min**	**95%CI max**	**20-years**	**95%CI min**	**95%CI max**	***p*-value**
**No IFN**	41	3	97.2	91.8	100	92.1	80.9	100	0.296
**IFN**	18	0	100	100	100	100	100	100	

IFN: interferon, No pts: number of patients, No Tx: number of thrombotic events, MFS: myelofibrosis-free survival

Figure S1: Univariate analysis for risk factors associated with thrombotic risk in the ET and PV patients.

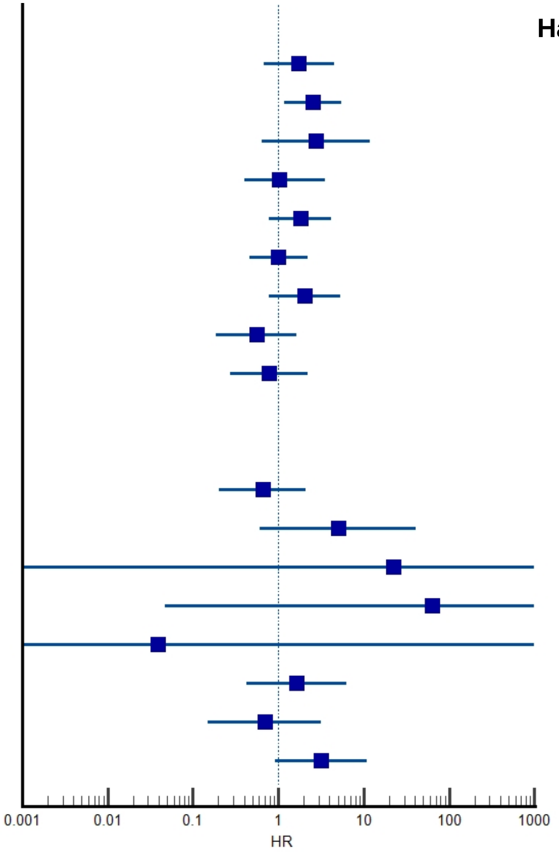

UNIVARIATE ANALYSIS – ET PATIENTS	Hazard ratio	(95% CI)	P value
Female (vs male)	1.747	(0.673-4.538)	0.252
JAK2 mutation (vs *CALR*, *MPL* or TN)	2.557	(1.182-5.529)	0.012
Absence of splenomegaly (vs splenomegaly)	2.750	(0.639-11.844)	0.174
HCT ≥45% (vs <45%)	1.023	(0.403-3.586)	0.741
WBC >11×10^9/L (vs <11×10^9/L)	1.809	(0.779-4.204)	0.168
PLT ≥1000×10^9/L (vs <1000×10^9/L)	1.013	(0.459-2.237)	0.975
Any thrombosis history (vs absence)	2.061	(0.789-5.380)	0.140
LDH elevated (vs not elevated)	0.554	(0.188-1.634)	0.285
Presence of any CVRF (vs absence)	0.779	(0.271-2.234)	0.642
**UNIVARIATE ANALYSIS – PV PATIENTS**			
Female (vs male)	0.654	(0.205-2.090)	0.474
Absence of splenomegaly (vs splenomegaly)	4.995	(0.614-40.624)	0.133
HCT ≥45% (vs <45%)	22.653	(0-5.2×10^7)	0.677
WBC >11×10e9/L (vs <11×10e9/L)	63.117	(0.047-8.4×10^4)	0.259
PLT ≥1000×10e9/L (vs <1000 × 109/L)	0.039	(0-2.2×10^3)	0.563
Any thrombosis history (vs TX history)	1.641	(0.428-6.294)	0.470
LDH elevated (vs not elevated)	0.691	(0.152-3.138)	0.632
Presence of any CVRF (vs absence)	3.164	(0.922-10.856)	0.067

CVRF: cardiovascular risk factors, ET: essential thrombocythemia, HCT: hematocrit, LDH: lactate dehydrogenase, PLT: platelet, PV: polycythemia vera, TN: triple-negative, WBC: white blood count

Figure S2: Univariate and multivariate analysis for risk factors associated with risk of myelofibrosis progression in the ET and PV patients.

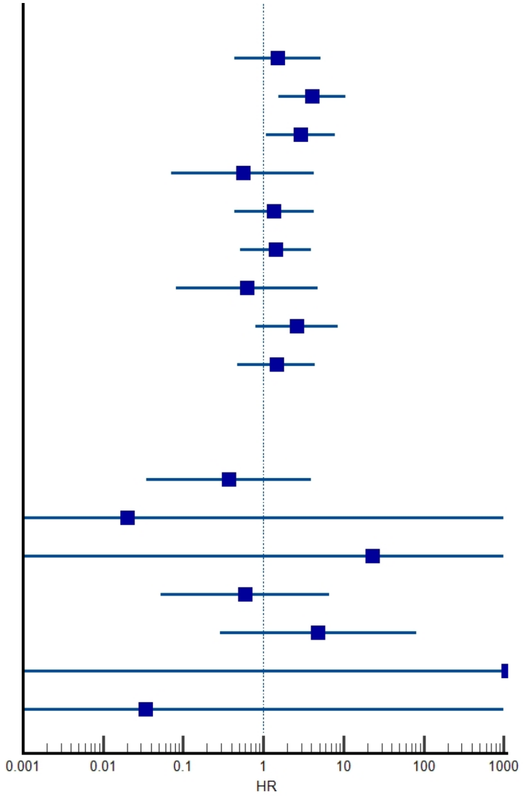

	Hazard ratio	(95% CI)	P value
**UNIVARIATE ANALYSIS – ET PATIENTS**			
Female (vs male)	1.515	(0.437-5.229)	0.513
CALR mutation (vs JAK2, MPL or TN)	4.065	(1.568-10.539)	0.004
Splenomegaly (vs absence of splenomegaly)	2.906	(1.098-7.694)	0.032
HCT ≥45% (vs <45%)	0.560	(0.072-4.350)	0.579
WBC >11×10e9/L (vs <11×10e9/L)	1.369	(0.434-4.316)	0.592
PLT ≥1000×10e9/L (vs <1000 × 109/L)	1.441	(0.522-3.982)	0.481
Any thrombosis history (vs TX history)	0.627	(0.083-4.731)	0.65
LDH elevated (vs not elevated)	2.622	(0.803-8.558)	0.11
Presence of any CVRF (vs absence)	1.462	(0.480-4.451)	0.504
**UNIVARIATE ANALYSIS – PV PATIENTS**			
Female (vs male)	0.375	(0.035-3.961)	0.415
Splenomegaly (vs absence of splenomegaly)	0.02	(0-2.5 × 10^3)	0.518
HCT ≥45% (vs <45%)	23.006	(0-7.5 × 10^{10})	0.779
WBC >11×10e9/L (vs <11×10e9/L)	0.595	(0.053-6.678)	0.674
PLT ≥1000×10e9/L (vs <1000 × 109/L)	4.83	(0.288-81.01)	0.274
Any thrombosis history (vs TX history)	1147	(0-9.1× 10^9)	0.385
Presence of any CVRF (vs absence)	0.034	(0-7.1 × 10^3)	0.589

CVRF: cardiovascular risk factors, ET: essential thrombocythemia, HCT: hematocrit, LDH: lactate dehydrogenase, PLT: platelet, PV: polycythemia vera, TN: triple-negative, WBC: white blood count

<u>Figure S3:</u> **Thrombosis-free survival according first-line cytoreductive drug. A) in ET patients. B) in PV patients.**

A)

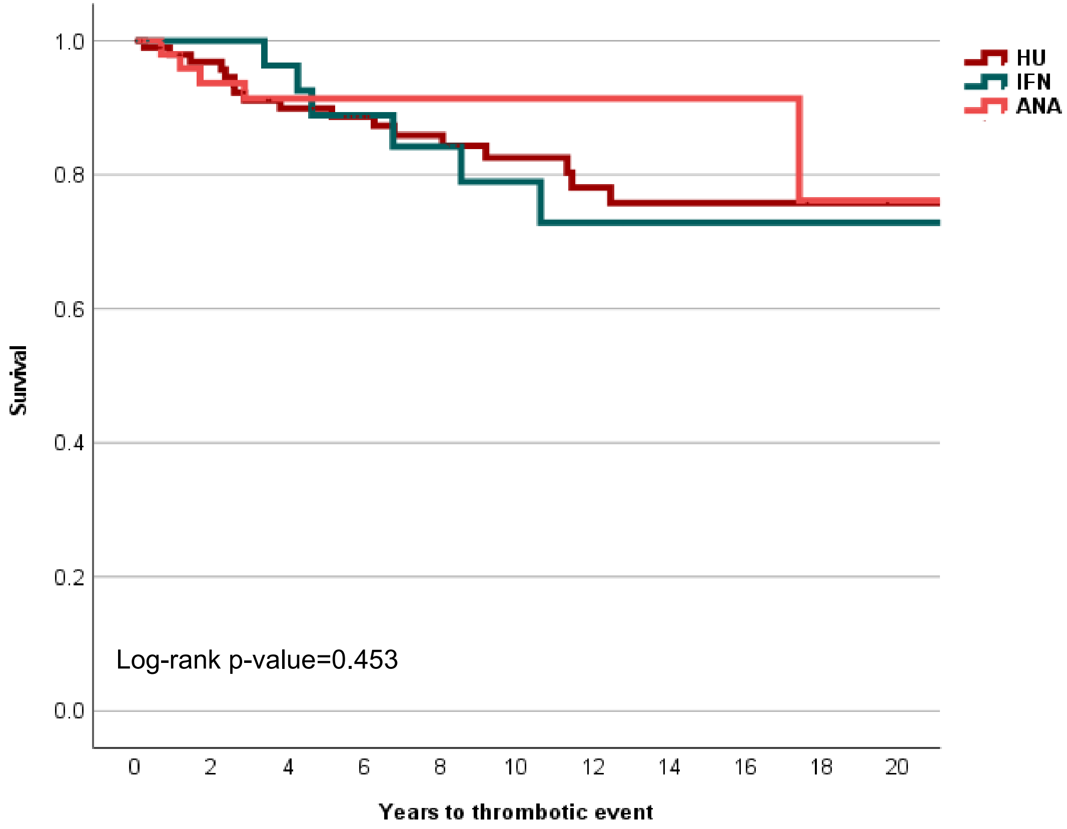

**No at risk :**											
HU	100	86	75	68	55	41	34	27	16	11	10
IFN	33	29	26	22	17	13	10	6	4	4	1
ANA	50	43	39	36	30	24	22	16	11	5	3

ANA: anagrelide, HU: hydroxycarbamide, IFN: interferon

B)

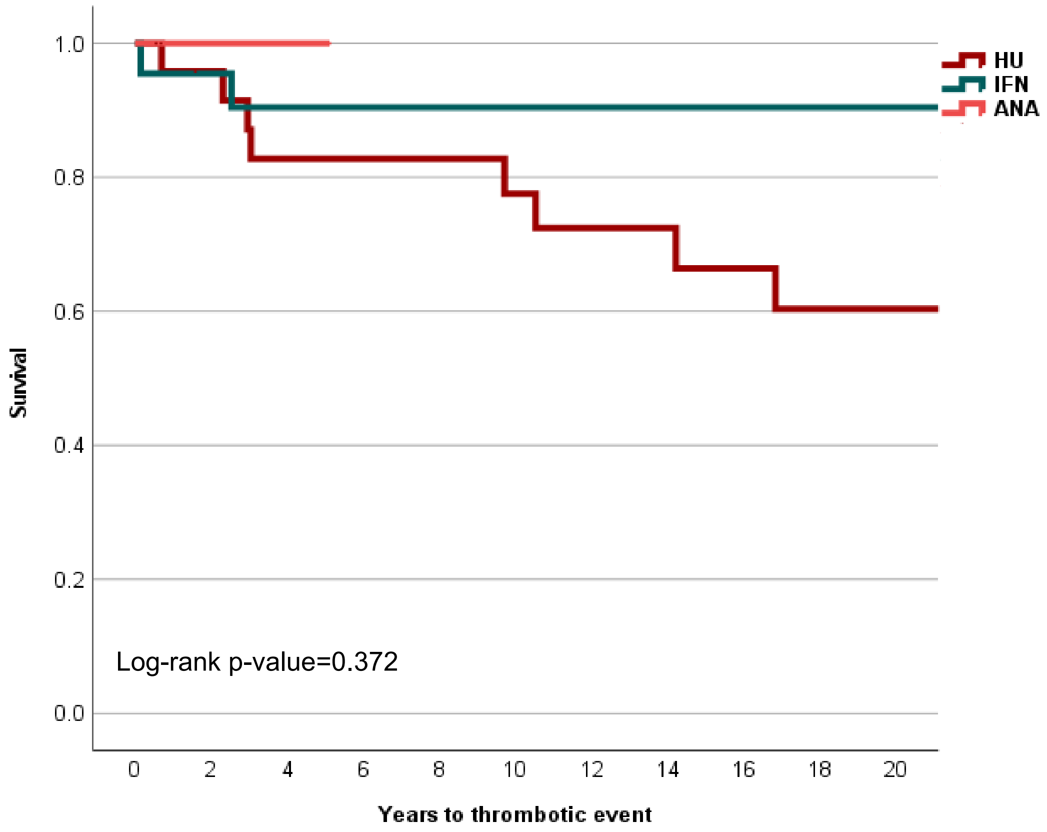

No at risk :

HU	26	22	19	18	17	15	13	12	11	7	5
IFN	22	21	18	18	13	9	5	5	4	2	2
ANA	1	1	1	0	0	0	0	0	0	0	0

ANA: anagrelide, HU: hydroxycarbamide, IFN: interferon

<u>Figure S4:</u> **A) Myelofibrosis-free survival according first-line management (use of interferon or not). A) in ET patients. B) in PV patients.**

A)

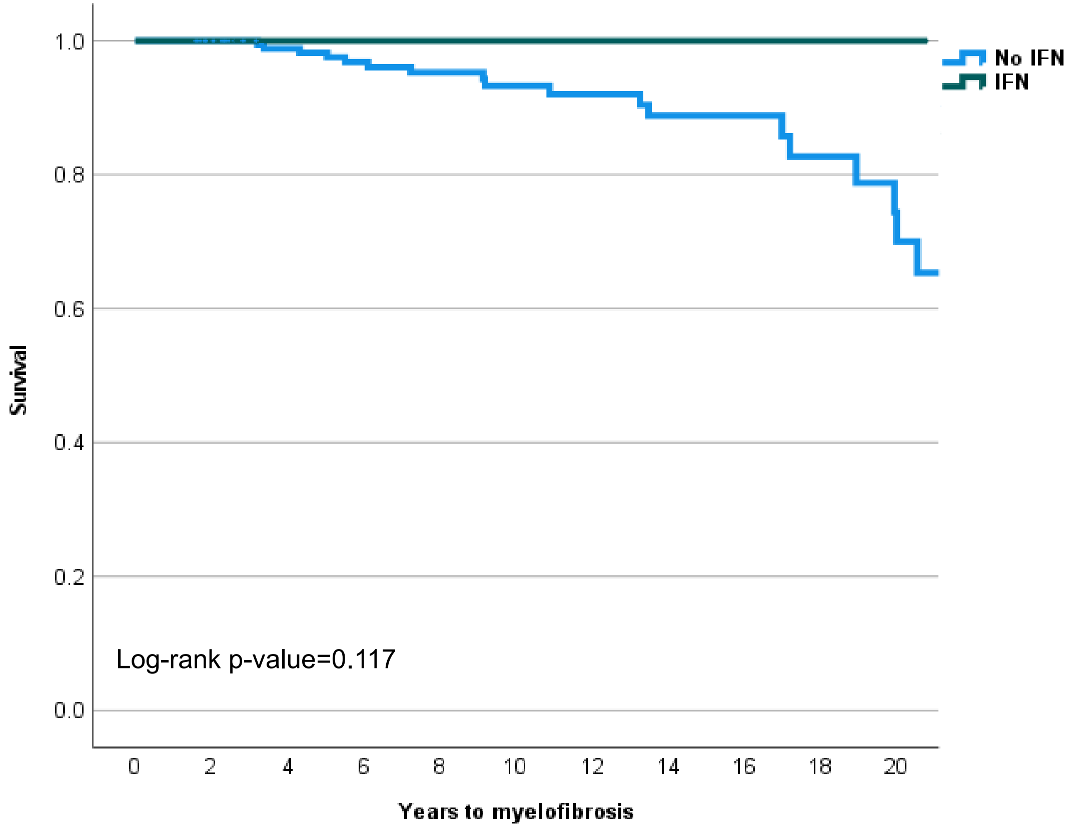

No at risk :

No IFN	194	187	162	132	107	82	69	49	36	23	17
IFN	24	24	22	21	18	15	13	8	6	5	1

IFN: interferon

B)

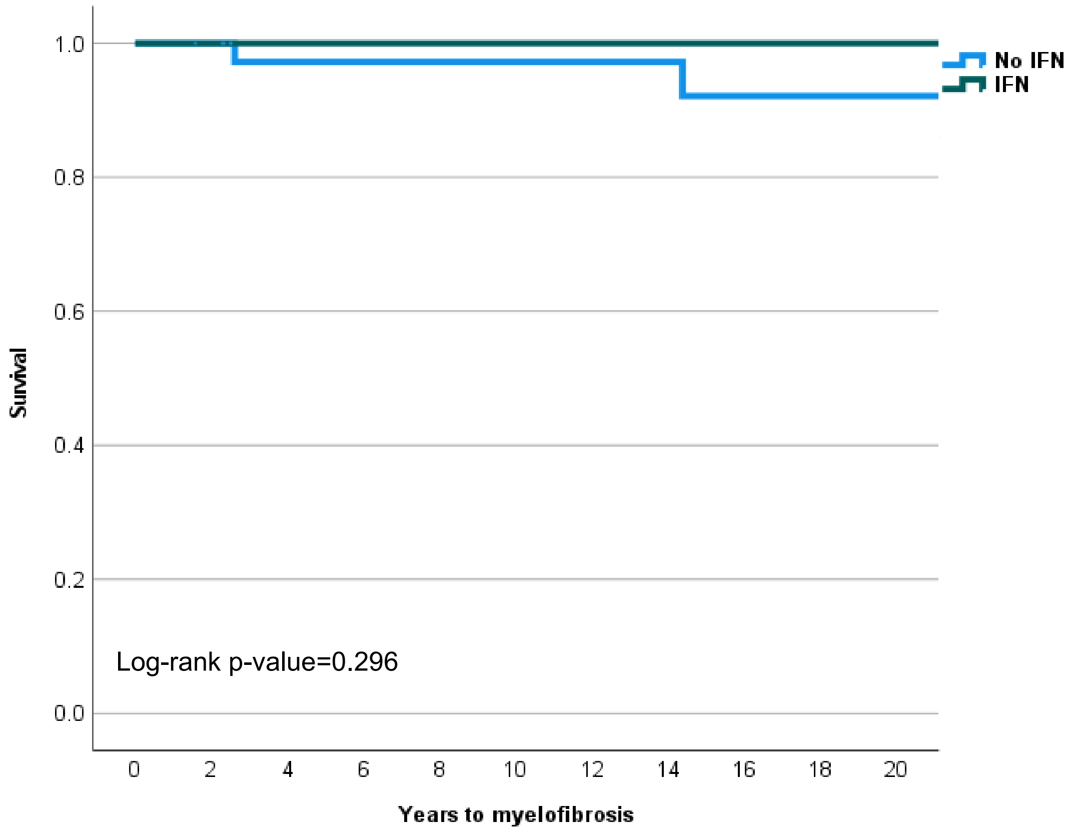

**No at risk :**											
No IFN	41	41	35	29	26	25	20	19	16	11	8
IFN	18	18	17	17	13	10	6	5	4	2	2

IFN: interferon
